# A Review of Advocate–Scientist Collaboration in Federally Funded Environmental Breast Cancer Research Centers

**DOI:** 10.1289/ehp.0901603

**Published:** 2010-07-09

**Authors:** Lori B. Baralt, Sabrina McCormick

**Affiliations:** 1 Department of Women’s, Gender and Sexuality Studies, California State University, Long Beach, Long Beach, California, USA;; 2 School of Public Health and Health Sciences, George Washington University, Washington, DC, USA

**Keywords:** collaborative research, community-based participatory research, environmental breast cancer research

## Abstract

**Background:**

The Long Island Breast Cancer Study Project was the first federally funded study of environmental causes of breast cancer. Although advocates were expected to participate in this study, the details of their participation were not adequately clarified in project guidelines, which resulted in confusion over their role in the project. The Breast Cancer and Environment Research Centers (BCERCs) are funded by the National Institute of Environmental Health Sciences and the National Cancer Institute; these centers continue to conduct research into environmental links to breast cancer and to clarify advocate–scientist guidelines for collaboration.

**Objectives:**

Practitioners in community-based participatory research (CBPR) are grappling with how to improve CBPR projects for all groups involved in breast cancer and environmental studies. The ever-growing body of literature on CBPR elaborates on a number of factors that make CBPR particularly challenging, specifically regarding partnerships between advocate and scientific communities. This study draws on CBPR principles to evaluate advocate–scientist collaboration in the BCERCs.

**Methods:**

We conducted surveys at BCERC annual meetings in 2005 and 2007 and 11 in-depth open-ended interviews with key stakeholders such as primary investigators within the centers to assess the perceptions of the advocates and scientists regarding collaboration between advocates and scientists who were engaged in CBPR studies.

**Results:**

We found that although participatory guidelines were a focus of BCERCs, underlying differences between advocates and scientists with regard to paradigms of scientific inquiry, priorities, and desired outcomes need to be addressed for more effective collaboration to take place.

**Conclusion:**

Our findings contribute to the broader CBPR literature by highlighting the role of underlying assumptions that may hinder the collaborative process and suggest the need for continued assessment research into participatory research projects on breast cancer and the environment.

The higher-than-average incidence of women with breast cancer in particular regions of the United States, especially in parts of New York, Massachusetts, and California, has motivated a push for scientific research into potential environmental causes of breast cancer (Brody et al. 2005; Brody and Rudel 2003; Brown et al. 2001, 2006; Eisenstein 2001; McCormick et al. 2004). Many women in these areas began seeking answers about why breast cancer rates seemed to be so high where they lived and advocated for increased scientific research into environmental factors that might explain the apparent spike in breast cancer incidence. Their persistence has generated a trajectory of research involving breast cancer advocates, many of whom have been largely responsible for new funding for research on potential environmental causes of the disease. Nevertheless, advocates have raised questions over the years about challenges to and effectiveness of participatory research structures and practices in these cases and others (McCormick et al. 2004).

The National Institute of Environmental Health Sciences (NIEHS) and the National Cancer Institute (NCI) created the Breast Cancer and Environment Research Centers (BCERCs) with the intention of adhering to effective participatory practices, although never specifically stating that they would adhere to community-based participatory research (CBPR) principles. CBPR projects have proven challenging, particularly with regard to advocate collaboration with scientists and researchers (Israel et al. 1998, 2005; Minkler 2005; Minkler and Wallerstein 2008). CBPR researchers and practitioners have found that the relationship between advocate and scientist participants in such projects is often strained because of a variety of issues including advocates’ lack of trust in scientists, advocates’ lack of training in and understanding of scientific research, and mutual frustration with the collaborative process (Israel et al. 1998, 2005; Minkler 2005; Minkler and Wallerstein 2008).

In this review, we assessed the collaboration between advocates and scientists to identify barriers to creating effective advocate–scientist collaboration in the BCERCs, which are the largest federally funded breast cancer and environment research projects to date. In this paper, we have defined advocates as Community Outreach and Translation Core (COTC) members who have pursued scientific answers and public health and policy outcomes regarding environmental links to breast cancer. Scientists are defined as BCERC participants trained in cell biology or epidemiology who are researching particular environmental impacts on the development of breast cancer. Using our findings, we established several recommendations to improve advocate–scientist collaboration [see Supplemental Material (doi:10.1289/ehp.0901603)]. This is especially timely, given that the NIEHS and the NCI will be funding a new multiyear breast cancer and environment research project that may continue much of the research that is being conducted by the BCERCs (NIEHS 2009). In addition, many of the advocates and scientists we interviewed expressed concern about the lack of evaluation of the current collaborative process and noted their desire to participate in future research if funding is available and if the collaborative process is improved.

## Background

The Long Island Breast Cancer Study Project (LIBCSP) was the first federally funded study of potential environmental causes of breast cancer. The study was started in 1992 and completed in 2002; it was a $31 million project that used multiple methodological approaches to assess which, if any, environmental exposures on Long Island might be linked to breast cancer [McCormick et al. 2004; NCI 2010; U.S. Department of Health and Human Services (DHHS) 2004]. The LIBCSP was designed to include advocate participation; however, the details of their participation were not adequately clarified in the funding mandate. This lack of clarity resulted in discontent regarding their role in the research process, which was largely expressed after the results of the study were disseminated. The advocates questioned whether the research methods and the variables selected by the scientists advanced the knowledge of possible carcinogeneity of environmental exposures (McCormick et al. 2004).

Although advocates fought hard to get the research project funded, in the end they felt that the researchers pursued their own research agendas and largely ignored their input. Because of the lack of specificity in the funding mandate, researchers and advocates did not have a clear idea as to when they were meant to engage with one another. For the LIBCSP, a group of organizations, collectively called the Long Island Breast Cancer Network, met regularly with the primary investigator (PI) of the study, which had some impact on the research (McCormick et al. 2004). However, because the terms of collaboration were not clearly defined, many activists felt that the variables used in the study were not in their interest. Ultimately, this discontent led to contention over the results of the LIBCSP that found a lack of statistically significant evidence for environmental links to breast cancer. Even as the LIBCSP was in its final stages of completion, officials at the NIEHS and some breast cancer advocates were anticipating the need for further study. In 2002, the NIEHS convened a brainstorming workshop that included researchers, clinicians, and advocates “to identify data gaps, bottlenecks and research needs” (BCERC 2009). The result of the workshop was a broad decision to “promote research that would characterize environmental exposures over the lifetime that could alter the risk of breast cancer development” (BCERC 2009). The NIEHS and the NCI then released a Request for Application (RFA) for the formation of multiple BCERCs that would be funded for a 7-year research cycle. Subsequently, the NIEHS and the NCI established the BCERC Network in 2003.

The proposed project was unlike the LIBCSP in several ways. First, it did not mandate the exact topics and geographic areas of study. Second, it created a much more formalized structure for advocate participation in the centers, particularly with regard to translation and dissemination of research findings. The new RFA mandated that there be two research components—a cell biology component to study environmental effects on the molecular structure and function of the mammary gland across the life span (e.g., puberty, pregnancy, menopause) and an epidemiological component to examine environmental and genetic determinants of age at puberty and the development of the mammary gland during puberty. The RFA stated that the primary goal was to establish a

. . . network of research centers in which multidisciplinary teams of scientists, clinicians, and breast cancer advocates work collaboratively on a unique set of scientific questions that focus on how chemical, physical, biological, and social factors in the environment work together with genetic factors to cause breast cancer. (NIH 2002)

In 2004, the NIEHS and the NCI released funding for four centers led by the University of California, San Francisco (UCSF; San Francisco, CA), University of Cincinnati (Cincinnati, OH), Fox Chase Cancer Center (Philadelphia, PA), and Michigan State University (MSU; East Lansing, MI). With the exception of MSU, which lacked the epidemiology component, the institutions used both research components. The RFA mandated that each center have a central participatory component called a COTC that would develop and implement strategies to translate the scientific findings of the center into information for the public and policy makers. According to the RFA, the activities of the COTC could include developing educational materials for children and adults about breast cancer and the environment; conducting environmental justice-related activities; conducting public awareness workshops, forums, and meetings with stakeholders to discuss issues related to breast cancer and the environment; and developing and evaluating novel approaches to disseminate research findings to interested parties.

Although requiring a participatory component, the RFA did not require the centers to use a CBPR approach. Advocates were included in the initial brainstorming workshop to discuss the potential topics of investigation; however, these advocates were not necessarily the same as those who would be involved in the particular centers, because the location of the centers were selected by the NIEHS and the NCI and were based on applications submitted by a PI and not by community advocacy organizations. The PI at each prospective center had to contact these organizations at the time of application and invite them to participate in the COTC, in the event that the center received funding. Thus, advocacy and community organizations participated in the studies (at least nominally) at the four funded centers [see Supplemental Material, Table 1 (doi:10.1289/ehp.0901603)], but community advocates were not necessarily involved in developing the initial center application or in deciding the research emphasis that would be used in a particular center. The COTCs of the UCSF, Fox Chase, and University of Cincinnati BCERCs consisted of representatives from advocacy organizations and academic researchers. At the MSU center, the translation and dissemination of research findings was led by professors from the Department of Communication; community and advocacy organizations were also included nominally in the center application and invited to attend the annual meetings. Based on our interviews with scientists and COTC members at MSU, however, these groups were otherwise not integrated into the project and serve as an example of how much discretion the centers had in terms of integrating advocates into the research process.

## Developing Effective CBPR

Over the past two decades, CBPR projects have played an increasingly significant role in addressing important health research questions. CBPR has been particularly relevant for health issues in which traditional biomedical approaches have proven insufficient (e.g., environmental health issues) (Brody et al. 2005, 2007; Brody and Rudel 2003; Brown et al. 2006; McCormick et al. 2004; O’Fallon and Dearry 2002). In the case of breast cancer, CBPR has proven particularly relevant in terms of examining potential gene–environment interactions in susceptibility to the disease (Brown et al. 2006; McCormick et al. 2004). Brown et al. (2006) and McCormick et al. (2004) found that before breast cancer advocates were involvded in research, breast cancer research focused almost exclusively on biomedical research and did not draw connections between the role of genes in breast cancer susceptibility and the potential role of environmental factors (Klawiter 2008). Despite the plethora of biomedical breast cancer research, susceptibility to breast cancer remains largely unexplained (Klawiter 2002). Many breast cancer advocates have focused on engaging scientists to understand how women are differentially genetically and environmentally susceptible to breast cancer (Shostak 2003). The BCERCs are a unique example of a participatory research model. For example, most CBPR projects have focused primarily on alliances between community advocates and public health researchers or epidemiologists (such as the LIBCSP), but the BCERCs also have included a cell biology component that has fostered transdisciplinary collaboration between biologists and epidemiologists as well as advocates.

The NIEHS has been a leader in promoting the use of CBPR in cases where community–scientist partnerships serve to advance the understanding of environmental health (Birnbaum 2009; Felix 2007). There are numerous challenges faced by these projects. CBPR researchers and practitioners have raised questions about the most effective forms of advocate participation and the various challenges that can hinder effective advocate–scientist collaboration in such projects (Israel et al. 1998, 2005; Minkler 2005; Minkler and Wallerstein 2008).

Researchers have shown that effective CBPR requires specific principles that guide the research process including articulating the values of the research, establishing mutual trust and cooperation between advocates and scientists, planning and designing collaborative research (Baker et al. 1999), educating community members regarding the science that is being used in a particular project, and forming fixed structures that provide formal power sharing (McCormick et al. 2004; Minkler et al. 2003). Thus, effective CBPR requires involving community members in formulating the research questions and the study design (Israel et al. 2005; McCormick et al. 2004) and often including them in the data collection process (Kinney et al. 2002). These principles provide the context for our research, because we are interested in examining how the BCERCs and future breast cancer and environment projects that use similar advocate–scientist collaborations can improve their collaborative processes.

One gap in the CBPR literature is that potential underlying issues within advocate–scientist research projects, such as each group’s assumptions about the scientific research process, their research priorities, and desired outcomes are not easily addressed by research guidelines. Thus, even when, as was the case with the BCERCs, collaborative research guidelines seek to clearly delineate a collaborative research design and process, underlying issues may be largely ignored. Although projects such as the LIBCSP and the BCERCs have general guidelines and requirements for advocate participation in the research process, other factors may inhibit advocate–scientist collaboration. For example, the norms assumed and attitudes possessed by scientists may be different than those of advocates (Minkler 2005). These issues may not be addressed through project guidelines and requirements. Therefore, we attempted to assess the underlying and less well-recognized issues that may be affecting participatory research projects like the BCERCs. Such issues include differences in attitudes and perceptions between advocates and scientists regarding inquiry paradigms and differences in concerns about and desired outcomes regarding environmental causes of breast cancer. These issues can be regarded as some of the principal challenges to creating participatory collaborations. Although the CBPR literature has addressed practices within such projects, it has rarely addressed the varied attitudes scientists and advocates bring to the table that could affect their opinions about the research process and outcomes (McCormick et al. 2004). This is a particularly important point in environmental breast cancer research, because history has shown that new projects often emerge from previous ones, many times involving the same scientists and advocates (McCormick et al. 2004).

## Methods

From 2005 to 2008, we collected qualitative and quantitative data to analyze perceptions of advocate–scientist collaboration and to better understand the challenges to advocate–scientist collaboration in the four centers funded by NIEHS and NCI. Our initial qualitative data included government, scientific, and advocate documents such as reports, research articles, and newsletters, which we used as background information regarding how advocate–scientist collaboration in these centers was supposed to proceed (Bay Area BCERC 2010; BCERC 2006b, 2006c; Cincinnati BCERC 2010; MSU BCERC 2010; NIEHS 2009; NIH 2002). We reviewed these documents for information regarding the roles of advocates and scientists in the BCERCs, the outcomes of meetings where scientists and advocates came together, and the perceptions of the advocates and scientists of the collaborative process (Bay Area BCERC 2010; BCERC 2006a, 2006b, 2006c; Cincinnati BCERC 2010; MSU BCERC 2010).

We also conducted ethnographic observations at two annual national BCERC meetings in 2005 and 2007, where we attended a variety of sessions in which biologists and epidemiologists presented their current research, and where COTC members presented strategies for disseminating research findings and community outreach stategies. Discussions between advocates and scientists were used to better understand the tensions expressed and relationships built between the participating groups. At these meetings, we conducted informal interviews using a short list of questions that were repeated in 2005 and in 2007 with both scientists and advocates regarding their perspectives on the progress of the research and on their own participation. These conversations were not transcribed or recorded or used directly as data. Rather, they were used to develop a sense of the collaboration between advocates and scientists to form our questions for the in-depth interviews (Berg 2004).

At the same two meetings, we administered surveys that we developed for this study. We developed parallel surveys, one for scientists and one for advocates. The advocate survey was entitled “Survey of Advocates’ Experience Collaborating with Scientists”and consisted of the same 22 questions in 2005 and in 2007. The scientist survey, entitled “Survey of Scientists’ Experience Collaborating with Advocates,” was also used in 2005 and 2007, with the exception of one question regarding priorities for research on potential environmental causes of breast cancer, which was omitted in 2007. The questionnaire was available for advocates and scientists to fill out anonymously at the conference registration table. We did not participate in selecting who would or would not fill out a survey. Rather, it was a self-selected group of advocates and scientists. Both surveys consisted of questions about what role they played in the center, whether they were involved in the center from the beginning, what they expected in terms of the outcomes of the center, whether they believed that there were environmental factors affecting breast cancer rates, what their top five priorities were regarding potential environmental causes of breast cancer, and where their views on environmental links to breast cancer came from. Advocates and scientists who were asked questions about what role they played in the center, whether they were involved in the center from the beginning, what they expected in terms of the outcomes of the center, whether they believed that there were environmental factors affecting breast cancer rates, what their top five priorities were regarding potential environmental causes of breast cancer, and where their views on environmental links to breast cancer came from. Because the question regarding priorities about potential environmental causes of breast cancer was omitted in the 2007 survey of scientists, when we draw on these data in our results, we do not make an argument regarding change over time, and the data are limited to the scientists surveyed in 2005 and advocates in 2005 and 2007. Additionally, we asked advocates about their previous work with scientists, their level of trust in scientists, and the type and duration of advocacy work they were involved in. Scientists were asked about previous work with advocates, expected and desired role of advocates in research, and ideas about potential benefits and challenges to working with advocates.

We combined the data for 2005 and 2007, giving us a total of 54 respondents (31 advocates and 23 scientists). Nineteen advocates and 13 scientists completed the survey in 2005; 12 advocates and 10 scientists completed it in 2007. Surveys were anonymous, but certain questions, such as which center they worked in, their particular scientific area of expertise, their place of employment, and their previous experience with particular collaborative research projects, were asked in such a way that allowed us to check whether any respondents who filled out the survey in 2007 had previously filled it out in 2005. Based on the answers we received, it did not appear that any of the respondents in 2007 had filled out the survey in 2005. At the 2005 meeting, about 160 participants attended, and about 220 persons attended the 2007 meeting. Both meetings were opened to the public, and participants consisted of a broader community than just advocates and scientists who were actively involved with the BCERCs. We compiled the data using Microsoft Excel spreadsheets (version 2007; Microsoft, Redmond, WA, USA), and we used it to analyze the views of the advocates and scientists about working with each other, about their views on priorities about potential environmental causes of breast cancer, and about their desired outcomes for the BCERCs. These data also helped inform our interview schedule by bringing to our attention particular areas where scientists and advocates seemed to diverge in their experiences collaborating with one another. We were able to delve further into these areas by developing interview questions about them.

Demographically, our sample (*n* = 54) was fairly representative of the key stakeholders in the BCERCS (i.e., the primary and coinvestigators on the science projects and the active members and leaders of the COTCs) (Table 1). Our sample overrepresented women scientists and advocates. Racially, it overrepresented Asian and African-American scientists and underrepresented white and Latino scientists. With regard to advocates, our sample overrepresented African-American advocates and underrepresented white and Latino advocates. Finally, advocates in our sample represented a broader range of education attainment than was present in the COTC leadership across the centers: 40% had a doctor of philosophy (PhD), 40% had a master of arts (MA) degree, and 20% had a bachelor of arts (BA) degree.

Between July and December 2008, we conducted eleven semistructured in-depth interviews that asked informants open-ended questions and probed wherever necessary to obtain data deemed useful for the research, with key advocate and scientist stakeholders, including at least one of each (advocate and scientist) from each of the centers, using purposive and snowball sampling techniques to gain further insight into the collaboration between advocates and scientists in the BCERCs and to confirm survey findings (Berg 2004; Straus and Corbin 1998). Purposive sampling involves selecting participants who are not necessarily representative of the larger population but who serve a specific need or purpose of the researchers (Berg 2004). We interviewed the PIs of the centers whenever possible, because they were involved in the centers from their inception. PIs could provide insight into the collaborative process between advocates and scientists, including if and when it had changed over time and if there were any long-term issues that should be addressed in future projects. We then used snowball sampling by asking the PIs who we should speak to in their center about issues of advocate and scientist collaboration. These sampling techniques contain particular biases and do not provide a representative sample of the population. The PIs may have referred us to advocates or scientists who had particularly strong feelings on the collaboration or to advocates who were more involved in the project than other advocates might have been.

The interviews at the MSU BCERC were conducted in person, and the interviews with participants of all other BCERCs were conducted by phone. All participants were asked about their involvement in their particular center (e.g., when they got involved, how they got involved, what their participation consisted of, how their participation was determined, in what context and how often they met with advocates or scientists, how they viewed the collaboration between advocates and scientists, and what the particular benefits or challenges were to advocate–scientist collaboration in their center). They were also asked questions regarding desired outcomes for the centers and views on potential environmental causes of breast cancer. Additionally, we asked them about whether they would choose to work on advocate–scientist collaborations in the future. We obtained written informed consent from all participants. We audiorecorded all interviews using a portable digital voice recorder and transcribed and coded them for themes of advocate–scientist collaboration, benefits and challenges to advocate–scientist collaboration, views on potential environmental causes of breast cancer, desired outcomes of the BCERCs, and the potential for future collaboration in research projects on breast cancer and the environment.

## Results

According to CBPR literature, the most frequently mentioned challenges to conducting effective CBPR are lack of trust in scientists by the advocates, perceived lack of respect for advocates by scientists, and mutual frustration (Minkler 2005). Our research on the BCERCs suggests that issues of trust, respect, and frustration may actually be symptoms of other issues, such as different underlying assumptions about scientific research, that can be improved by clearly defining participatory research for all participants at the onset of the research project. The findings from our survey and the in-depth interviews suggest that in the case of the BCERCs, there was a lack of understanding of and training in CBPR as an alternative inquiry paradigm (Israel et al. 1998), particularly among the biological scientists and some of the advocates who were brought into the project. The survey results and the interviews further demonstrate that the lack of understanding or training in CBPR was exacerbated by divergent assumptions and desired outcomes regarding environmental causes of breast cancer. The interviews highlighted the fact that the lack of understanding of or training in CBPR and divergent assumptions and desired outcomes regarding environmental causes of breast cancer were not consistent across the BCERCs. Rather, differences between BCERCs accentuated problems regarding training in CBPR, divergent assumptions and desired outcomes, while demonstrating the potential for overcoming these issues through increased understanding of and training in CBPR. The following results draw primarily from our survey and interview data. The interviews confirmed our survey findings and also provide a glimpse into the differences between the centers. We present our results in the two main categories of challenges to participatory research in the BCERCs: *a*) lack of understanding and training in CBPR as an alternative inquiry paradigm, and *b*) divergent prior assumptions and desired and expected outcomes regarding environmental causes of breast cancer.

## CBPR: An Alternative Inquiry Paradigm

A paradigm regarding how knowledge is produced and who can produce knowledge underlies all scientific research projects (Minkler 2005). The positivist paradigm remains dominant in much scientific research, emphasizing objective knowledge that is separate from the knower and that can be uncovered only through a scientific method of inquiry that is neutral and free of bias. Many scientists and nonscientists uphold this method of inquiry. CBPR challenges this paradigm by contextualizing scientific research within particular communities, including and legitimizing knowledge, understandings, and priorities of the advocates regarding issues by which they are personally affected (Bidwell 2009; Israel et al. 1998; Minkler 2005; Minkler and Wallerstein 2008). This alternative inquiry paradigm, therefore, challenges the understanding of many scientists and advocates of what science is, how knowledge is produced, and who can participate in producing knowledge.

In the case of the BCERCs, we found that the lack of understanding of and training in CBPR as an alternative paradigm hindered the collaboration between advocates and scientists, leading to mutual frustration. Although all the scientists agreed that advocates made positive contributions to science, many (40% in 2005 and 33.3% in 2007) also felt that the involvement of advocates hindered scientific research. The decline from 40% of scientists in 2005 to 33.3% in 2007 may suggest that over time, as scientists collaborate with advocates, they come to appreciate contributions made by the advocates. It may also suggest that as some advocates noticed the frustration of the scientists, they withdrew from the process and became less involved. In this case, scientists may have found advocates to be less of a hindrance simply because they were participating less as the project progressed. Alternatively, it is possible that this relatively small change in percentage may simply be the result of having different scientists fill out the survey in 2005 and 2007. Whatever the case may be, the fact that between 33% and 40% of scientists reported feeling that the advocates’ participation hindered scientific research suggests that a proportion of scientists did not entirely embrace the alternative inquiry paradigm of collaborative research.

When interviewed, all but one of the scientists reported that advocate involvement “slowed down” scientific research. All of the advocates interviewed were aware of the fact that the scientists felt this way. As one public health advocate participating in the UCSF BCERC reported:

The mouse model is not my language at all. So I sit on these conference call meetings and have a much harder time trying to interject and ask for clarification, because it’s sometimes so far past the point where I even know what they’re talking about that it’s hard to know what to ask for clarification on. There’s always a struggle in trying to work as an equal research partner with scientists and researchers, when you’re representing the community and more of the public health angle. Because it does slow the process down, there needs to be time spent on allowing for that understanding so that true collaboration can actually take place, and I think a lot of scientists are not used to working that way for sure, and haven’t developed the patience to do so.

Three of the five scientists who were interviewed also commented on the watering down of the science that was often required to include advocates in the annual meetings. As one scientist at the MSU BCERC stated:

They [the annual meetings] are often superficial and the hard science that I might share with a scientist from another institution is going to happen at either the special science sessions, which we run at a 6-month interval before the regular meeting or after the regular meeting. We have a little more basic science discussion at those but even there . . . for communication reasons, you’re not getting into as much hard science. . . . On the other hand, naturally the advocates don’t want to be excluded from anything. They want to hear everything even though sometimes it can get over their heads a little bit.

In both the surveys and interviews, scientists frequently expressed views that demonstrated a tension between trying to embrace a collaborative research process while still being grounded in a positivist paradigm. Because working with advocates is often new to scientists, the collaborative process can lead to frustration, particularly if it is perceived as a hindrance to positivist science rather than as an alternative research approach. Based on survey results, scientists often viewed advocates as making useful contributions to science by successfully advocating for funding for the BCERCs. Additionally, in one case, researchers were prompted to investigate effects of perfluorooctanoic acid exposures on mammary gland development in mice because of community concerns about environmental contamination from a local chemical plant. The researchers found preliminary evidence of effects on mice that they are continuing to evaluate.

At the same time, survey results indicated that when it came to the actual implementation of scientific research and communicating research for it to be accessible to advocates, scientists often became frustrated. Without training in and commitment to the CBPR process as an alternative approach to scientific inquiry, scientists will continue to feel this type of frustration, as the inclusion of advocates in science is not consistent with a positivist approach (Israel et al. 1998).

Many advocates also expressed views that demonstrated a tension between trying to embrace a collaborative research approach while still being grounded in a positivist paradigm. In the case of advocates, this was often expressed as feeling like they did not know what their role should be or, as mentioned previously, feeling like they were hindering or interrupting the scientific process. According to the advocates interviewed at the University of Cincinnati and Fox Chase BCERCs, they were primarily responsible for recruiting and retaining participants for the epidemiological research projects at the centers and did not have much prior experience with or training in participatory research. Based on interviews with two advocates working with the UCSF BCERC, many of the Bay Area advocates were more experienced with CBPR because of prior experience with advocate and researcher collaborations and therefore were able to negotiate their role in the BCERC. In the MSU BCERC, however, perhaps because of the lack of the epidemiology component and the significant role played by faculty from the Department of Communicaton in translation and dissemination, advocates were very disconnected from the center. Interviews with both scientists and advocates from the MSU BCERC demonstrated a lack of consistent contact with one another. Although the two scientists interviewed continued with their research and did not seem concerned by the lack of participation by the advocates, one of the advocates interviewed reported feeling uncertain about what her role in the project should be. As she put it,

Initially, we got involved because they needed advocate support for the application and I wrote a letter on behalf of our organization in talking about the value of this type of research and information and what we might be able to offer as far as potentially helping to get individuals involved. After that, we were all brought together, advocates and scientists, at different times for meetings, a couple times a year, but other than that we really fell out of the loop. I know they are doing great research, but we really aren’t part of it and the other advocacy groups at our center have fallen away as well. I don’t even remember who all they were to begin with anymore. But we are here to help if they need us.

Because a number of advocates did not view themselves as integral to the scientific inquiry process, which they were supposed to be according to the RFA, they did not pursue closer engagement, but instead waited for scientists to let them know when they were needed, demonstrating a deference to both scientists and the positivist inquiry paradigm.

Differences between BCERCs highlighted these issues while demonstrating the potential for overcoming them. The Bay Area BCERC provides an exemplary model for the project with regard to embracing CBPR as an alternative inquiry paradigm largely because of the experienced environmental breast cancer advocates who had training and previous experience with CBPR projects. Unlike Cincinnati, East Lansing, or Philadelphia, the San Francisco Bay Area has a long history of environmental breast cancer activism spurred by the higher than average rates of breast cancer in the area (Klawiter 2008). Many breast cancer activists in the area have collaborated with scientists, and there are a number of organizations that focus specifically on connections between breast cancer and the environment, which was not the case with the other centers (Klawiter 2008). As a result, even if scientists in the Bay Area BCERC had no experience with the alternative research paradigm, the advocates were able to negotiate their role in the BCERC, as demonstrated by the comments of this Bay Area COTC member:

[Our COTC] consists of people who, like us, have actually been in research projects for a long time, who understand the concepts, can demand what we want, and there are also members of our COTC who have been actively involved in pushing legislation through the state of California to ban chemicals, chemicals like phthalates, the same ones we’re studying [in the BCERC], phthalates and bisphenol A. So it’s a politically sophisticated as well as a scientifically sophisticated COTC. And then certainly people who are involved in public health and the breast cancer survivors are very articulate, politically active survivors.

Bay Area advocates differed from advocates from the other centers in that their previous experience working with scientists had provided them with training in basic science. This allowed them to participate more actively in the BCERC. Although the Bay Area BCERC consists of advocates who are well practiced in CBPR and have therefore been able to implement a more collaborative advocate–scientist model, their success indicates that other BCERCs also have the potential to do so with additional advocate training in CBPR and basic science.

## Priorities and Desired Outcomes Regarding Environmental Causes of Breast Cancer

Advocates and scientists bring different conceptions of and motivations for research to the table. In many research projects, these differences are reflected in their beliefs about the topic being studied (Minkler 2005). Our data indicate that advocates and scientists entered collaborations with different perspectives on potential environmental causes of breast cancer. All of the advocates and scientists surveyed reported that they believed environmental factors contribute to breast cancer. Further questioning, however, clearly showed that each group conceptualized “environment” quite differently. Answers to the survey question regarding the top five priorities among potential environmental causes of breast cancer reflected qualitative differences between the conceptions of advocates and scientists of potential environmental causes of breast cancer. We coded the responses into exogenous (e.g., pesticides, toxicants, and phthalates) and endogenous (e.g., diet, stress, obesity) environmental causes of breast cancer. We acknowledge that the terms exogenous and endogenous are not completely accurate because diet, stress, and obesity are affected by the external environment and by social context and are not solely individual or genetic issues. Nevertheless, the terms allow us to separate out a notable difference between what scientists and advocates tend to be concerned about within the broad arena of potential environmental factors. Although all of these factors can be included within a broad definition of environment, exogenous factors are more fully outside of an individual’s control; endogenous factors, although also affected by environmental and social factors, are generally classified as lifestyle issues and are often seen as being more within an individual’s control. Endogenous factors are also better studied with regard to their relationship to cancer risk. For respondents with lists that included both exogenous and endogenous causes, we added a combination category.

Although the RFA for the BCERCs provided a very broad definition of environment, advocates participating in the BCERCs were concerned primarily with exogenous environmental causes of breast cancer. According to the director of one of the BCERCs,

The BCERC project defines the environment in a lot of different ways, the food you eat, where you eat, the kind of food you eat, whether it’s organic or not, the air that you breathe, and the pesticide exposure, and, by far, the pesticide exposure is the thing that the advocates are most concerned about.

**Figure 1:**
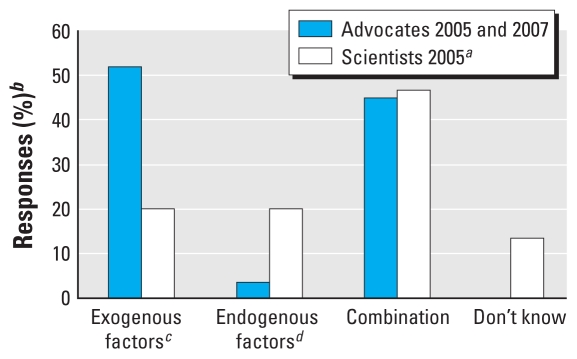
Priorities for research on potential environmental causes of breast cancer. ***a***Data not available for scientists 2007. ***b***Survey results for advocates from 2005 and 2007 were combined. Responses are reported as percentages (i.e., advocates who answered exogenous factors divided by the total number of advocate responses). ***c***Exogenous factors included, but were not limited to, pesticides and herbicides, man-made chemicals, car emissions, chemicals in cosmetics and deodorants, contaminated food sources, chemicals in cleaning products, phthalates, bisphenol A, and benzene. ***d***Endogenous factors included, but were not limited to, diet and nutrition, hormone replacement therapy, lack of exercise, obesity and body size, stress, smoking, and alcohol consumption.

When interviewed, advocates noted that although much scientific research had been conducted on individual risk factors for breast cancer such as smoking, high-fat diet, alcohol consumption, and stress, very little research had been conducted on environmental toxicants as they relate to breast cancer. The majority of advocates surveyed and interviewed expressed hope that the BCERCs would begin to fill this gap in existing breast cancer research. More than 95% of the advocates surveyed expressed concern about solely exogenous environmental causes of breast cancer or a combination of exogenous and endogenous causes. In contrast, scientists tended to conceptualize environmental causes of breast cancer as a combination of exogenous and endogenous environmental factors (Figure 1). For advocates, particularly advocates who have had breast cancer, there is a strong sense of certainty as well as urgency regarding environmental causes of breast cancer. According to one advocate affiliated with the UCSF BCERC, who had never had breast cancer, but had experience working on environmental links to breast cancer:

I think clearly the environment plays a role in breast cancer. Nobody can deny that there’s an environmental component to breast cancer, nor that there is a gene/environmental interaction that explains why some people are exposed to certain things and develop a disease and other people are exposed to the same thing and don’t develop a disease. So . . . I feel that you certainly cannot ignore the environment. But I also recognize how complex breast cancer is.

This environmental breast cancer advocate had years of experience working in the field of breast cancer and environmental exposure research, as well as working collaboratively with scientists using CBPR, and understood the challenges involved in researching the complexities of gene–environment interactions. Other advocates without this prior experience were often frustrated by what they perceived to be the tentative views of scientists on environmental causes of breast cancer.

Based on survey results, although 75% of advocates cited lay evidence/personal experience or a combination of lay evidence (e.g., observing higher rates of breast cancer in a particular geographic area) and scientific research as the basis for their certainty about environmental causes of breast cancer, scientists presented a much more tentative view of environmental links to breast cancer and cited scientific evidence as the basis of their cautious views regarding potential environmental links to breast cancer. The tentative nature of scientific research and the views of the scientists are in stark contrast to the certainty and urgency expressed by advocates, which has the potential to lead to frustration and conflict regarding expected outcomes of the BCERCs.

When asked about potential outcomes from the BCERCs, the scientists interviewed emphasized the complexity of the issues, the early stage of the research into environmental factors in breast cancer, and the fact that answering some of these early research questions just leads to more questions and not necessarily any translatable public health messages. Advocates, on the other hand, expressed their desire for scientific outcomes that can be translated into recommendations and actions within their communities to reduce breast cancer risk. As one advocate stated:

It’s just a little overwhelming when you think about how you can look at this issue and really actually make an impact on what we know and, more importantly, what actions we can take to decrease breast cancer occurrence everywhere and decrease personal risk. When you think about this project, and then you think about all the work that’s being done, and then you think about what we’re really studying, it’s just a drop in the bucket. So that can be kind of frustrating to communities in crafting messages. And I think people get really frustrated looking at environmental issues in relation to breast cancer . . .

As this advocate states, the potential for frustration and disappointment is always present if definitive outcomes and translatable results are not achieved.

## Discussion

A recent study that evaluated the participatory approach of the San Francisco Bay Area BCERC COTC found that the successful inclusion of community members and advocates in the project led to important benefits including improved relationships among diverse stakeholder groups, knowledge creation, and increased community support of the research (Van Olphen et al. 2009). At the same time, they found that certain atypical features of the collaboration (e.g., the basic biology component of research, involvement of general community members and experienced activists) resulted in different levels of participation among stakeholders (Van Olphen et al. 2009). Our study furthered the analysis of advocate–scientist collaboration in the BCERCs by assessing the perceptions of the collaboration by advocates and scientists, conceptions of knowledge generation, and priorities and desired outcomes regarding potential environmental causes of breast cancer across the centers, which allowed for a broader analysis of the BCERC project.

Our research demonstrated that the involvement of the advocates, although mandated by the RFA, varied across centers, with the Bay Area BCERC exhibiting the best example of successful collaboration. The Bay Area BCERC, therefore, although an important case to study on its own, is particularly useful in the context of all of the centers, as it sheds light on factors that may contribute to improved collaboration. Although the specification of advocate participation in the centers appears to be an improvement over the LIBCSP in terms of mandating the existence of the COTC at each center, the inclusion of advocacy organizations in the RFA, and annual meetings in which advocates and scientists came together, our research has shown that even these guidelines require refinement for improved advocate–scientist collaboration. Although project guidelines often specify certain aspects of advocate–scientist collaboration (e.g., in what context and how often they are supposed to meet with each other), we found that underlying differences between advocates and scientists played a role in the collaboration and need to be addressed in future projects.

As with the assessment of the needs of the community partners of the Tampa Bay Community Cancer Network by Gwede et al. (2009), we sought to assess the priorities of advocates involved in the BCERCs. Like Gwede et al. (2009), we found that although advocates are interested in scientific research, they tend to emphasize applied or community relevant priorities and goals. Gwede et al. (2009) concluded that “academic partners must frame and operationalize research objectives in ways that respect and achieve this goal—research that is important to and benefits the community directly.” Although Gwede et al. (2009) focused specifically on the priorities and expectations of the community partners, we examined the priorities and desired outcomes of advocates and of scientists. By including the perspectives of both advocates and scientists, it became clear that examining the relationship between breast cancer and the environment can have different meanings to different stakeholders; this issue needs to be addressed at the initial stage of the collaborative process to avoid disappointment on the part of the advocates at the conclusion of the research.

Based on our findings, the BCERCs have embraced elements of CBPR to varying degrees. Israel et al. (1998) suggested that there are three broad, nonmutually exclusive categories of challenges to participatory research projects, namely, partnership-related issues; methodological issues; and broader social, political, economic, institutional, and cultural issues. Partnership-related issues connected to lack of trust and respect between community members and researchers have been particularly well documented in CBPR literature (Israel et al. 1998). Israel et al. (1998) recognized that conflicts associated with differences in perspective, priorities, assumptions, values, beliefs, and language also pose potential problems to participatory research projects. Our research adds to this area of research on partnership-related issues by examining the underlying norms assumed and attitudes possessed by advocates and scientists participating in the BCERCs. Our findings suggest that these types of partnership issues are not necessarily addressed by guidelines that call for inclusion of advocates in the research process. These underlying norms and attitudes must be addressed at the outset of the collaborative process and throughout the process, if needed, to achieve effective collaboration. There are ways to structure the early stages of a project to ensure this outcome.

Evaluations of CBPR projects often emphasize aspects of the collaborative process or issues surrounding trust, respect, and frustration experienced by the advocate and scientist participants. Our paper has focused on the underlying issues of competing inquiry paradigms and divergent priorities and desired outcomes. Our research suggests that these underlying issues have affected the collaborative processes of the BCERCs and have contributed to frustration among both advocates and scientists. Because many advocates and scientists in the BCERCs lacked prior experience with collaborative research as an alternative inquiry paradigm, many participants, particularly advocates, have struggled to figure out what their role is in the project. Both scientist and advocate participants expressed frustration regarding the lack of clearly defined roles for the advocates in the project. Additionally, divergent prior assumptions and desired/expected outcomes regarding environmental causes of breast cancer have also led to advocate frustration. These issues must be addressed if advocate–scientist collaboration in the BCERCs is to be improved. Given the commitment of the NIEHS and the NCI to fund new environmental breast cancer research projects (BCERPs), it is crucial that these issues are addressed sooner rather than later.

Furthermore, although we noted that the RFA for the BCERCs did not specify that the centers adhere to CBPR principles, our findings indicate that advocate participation was shaped by the community in which the center was located. Israel and colleagues discussed the critical distinction between CBPR that “emphasizes conducting research *in a community* as a place or setting” and CBPR that emphasizes “conducting research *with a community* as a social and cultural entity with the active engagement and influence of community members in all aspects of the research process” (our emphasis) (Israel et al. 1998, 177). The Bay Area BCERC provides an example of a center that was able to engage with a community as a social entity, largely because the Bay Area has an established history of environmental breast cancer activism. Many of the other centers collaborated with community and advocacy organizations that did not focus on environmental breast cancer issues or, in some cases, on breast cancer at all. With the upcoming funding of the BCERPs, the NIEHS and NCI may consider focusing more on the community where the centers will be located to foster a more community-based approach to the project.

## Conclusion

This study has particular strengths and limitations worthy of mention. Although we support CBPR as an orientation to research that seeks to make outcomes more relevant to the community in question, our research approach was not consistent with CBPR principles. Rather, we evaluated the BCERCs from a sociological outsider perspective. It is important to clarify that advocates and scientists were involved in our research as participants, but they were not collaborators in our study. That said, we found that many participants, including advocates, scientists, and PIs, supported our “outsider” research into the advocate–scientist collaboration within the BCERCs, as they expressed concerns about the lack of evaluation of the research process. Additionally, because we were outsiders, we may have been able to elicit more candid views from advocates and scientists regarding their perceptions of the collaboration. Upon publication, we will send a copy of the article to all of the center PIs and interview participants. Future evaluations of the BCERCs, and participatory research projects more generally, however, would also benefit from research from within, which may generate different perspectives.

Our data were also limited to two survey years and a small number of interviews; there could be a greater number and range of conclusions with expanded data collection. We used a self-selected survey sample, that is, those who chose to fill out the survey may have been somehow different from those who chose not to. Our interviews were based on purposive and snowball sampling techniques, which led to interviews with advocates and scientists who were mostly well connected with the centers, with the exception of one advocate from the MSU BCERC who acknowledged not being well connected to the project. The perspectives of these advocates and scientists may be qualitatively different from other advocates and scientists who were not interviewed. Our findings reflect only our sample of possible respondents and therefore may not be generalizable to all center advocates and scientists. Furthermore, our findings may not be generalizable to advocates and scientists involved with other types of CBPR projects.

Despite the limitations of this study, it did generate findings that we find relevant and potentially beneficial for future advocate–scientist collaborations in environmental breast cancer research projects as well as in other similar studies. Recommendations based on our findings are available in Supplemental Material (doi:10.1289/ehp.0901603). This is a valuable contribution looking forward, given the inherent embeddedness of environmental health concerns in particular communities. It is our hope that other center-based environmental health programs will benefit from these findings. This research has added to the discussion about participatory research by highlighting the underlying issues that often remain unaddressed by project guidelines that must be addressed to foster more productive participatory research collaborations.

## Figures and Tables

**Table 1:** Characteristics of scientists^a^ and COTC members and advocates, by center.

	MSU		Fox Chase		University of Cincinnati		UCSF		Total *n* (%)	
	Scientists	COTC/advocates^b^	Scientists	COTC/advocates	Scientists	COTC/advocates	Scientists	COTC/advocates	Scientists	COTC/advocates
Sex										

Women	1	2	4	2	3	5	7	4	15 (62.5%)	13 (86.7%)
Men	2	1	2	0	2	1	3	0	9 (37.5%)	2 (13.3%)

Race										

White	3	3	5	1	5	6	9	4	22 (91.7%)	14 (93.3%)
Latino	0	0	1	1	0	0	0	0	1 (4.1%)	1 (6.7%)
Asian	0	0	0	0	0	0	1	0	1 (4.1%)	0

Education										

BA	3	0	0	0	0	3	0	0	0	3 (20%)
MA	0	0	0	1	0	1	1	4	1 (4.1%)	6 (40%)
PhD	0	3	4	1	4	2	9	0	20 (83.3%)	6 (40%)
MD	0	0	2	0	1	0	0	0	3 (12.5%)	0

MD, doctor of medicine.

a The category of scientist includes both laboratory scientists and epidemiologists. These numbers are based on the primary and coinvestigators on the laboratory science and epidemiology components of the BCERCs. In most cases, there were additional scientists working on these projects, but the information reported here is based on the main participants who were listed on the BCERC Web site (BCERC 2006a).

b The category of COTC/advocates includes the COTC member leadership. In most cases, there were additional advocates working with the COTC, but the information reported here is based on the main participants who were listed on the BCERC web site (BCREC 2006a).
